# Elective laparoscopic colectomy in a patient 3 weeks after coronavirus disease 2019 infection: a case report

**DOI:** 10.1186/s13256-021-02877-4

**Published:** 2021-05-18

**Authors:** Yuki Tateno, Kimito Harada, Fumiki Okamoto, Hideo Katsuragawa

**Affiliations:** Department of Surgery, Tama-nambu Regional Hospital, 2-1-2, Nakazawa, Tama, Tokyo, Japan

**Keywords:** COVID-19, Colorectal cancer, Laparoscopic colectomy

## Abstract

**Background:**

According to previous reports, surgery is not recommended until at least 4 weeks after the symptoms of coronavirus disease 2019 resolve. However, strong evidence has not been established regarding the optimal timing and preoperative examination for elective laparoscopic colectomy for colorectal cancer in individuals with a previous coronavirus disease 2019 infection.

**Case presentation:**

A 63-year-old Asian man underwent elective laparoscopic colectomy for sigmoid colon cancer 3 weeks after asymptomatic coronavirus disease 2019. The postoperative course was good, and none of the surgical staff was infected with coronavirus disease 2019.

**Conclusion:**

In this patient infected with coronavirus disease 2019 within 4 weeks of surgery, preoperative venous ultrasound of the lower extremities and a chest computed tomography scan were useful examinations for ensuring a safe surgical procedure for the patient and the staff. Surgery within 4 weeks may be possible with careful selection of cases based on thorough preoperative examination. This report may contribute to the development of a consensus on performing safe elective colectomy for colon cancer in persons previously infected with coronavirus disease 2019.

## Background

Since the beginning of the coronavirus disease 2019 (COVID-19) pandemic in early 2020, the recommendation has been to postpone elective laparoscopic colectomy for colon cancer as long as possible because of the need to devote limited equipment and medical staff to the management of COVID-19, the high risk of postoperative complications in patients with colon cancer and COVID-19, and the risk of infecting staff and other patients [1–3]. Elective laparoscopic colectomy can be postponed for up to 3 months when colon cancer is in stage III or lower, considering that surgical treatment would be difficult in situations of tumor progression [4]. According to previous reports, surgery is not recommended until at least 4 weeks after the symptoms of COVID-19 resolve to be considered safe [5, 6]. However, there has been no strong evidence on how to resume postponed elective laparoscopic surgery. In addition, evidence of preoperative examination for safe perioperative management has not been established. Considering the high prevalence of COVID-19 worldwide, strong evidence the strategy for elective laparoscopic colectomy for patients previously infected with COVID-19 should be established immediately.

## Case presentation

A 63-year-old Asian man who had positive fecal occult blood underwent colonoscopy, which showed an ulcer-shaped cancerous lesion in the sigmoid colon (Fig. 1). Pathologic examination revealed moderately differentiated tubular adenocarcinoma. Based on the absence of obvious lymph node and distant metastases on computed tomography (CT), the assessment was clinical stage II colon cancer, and elective laparoscopic colectomy was planned for 2 months later. Routine preoperative blood tests, electrocardiogram, and chest X-ray showed no abnormal findings, except for mild diabetes mellitus (glycated hemoglobin of 6.4%).

**Fig. 1:**
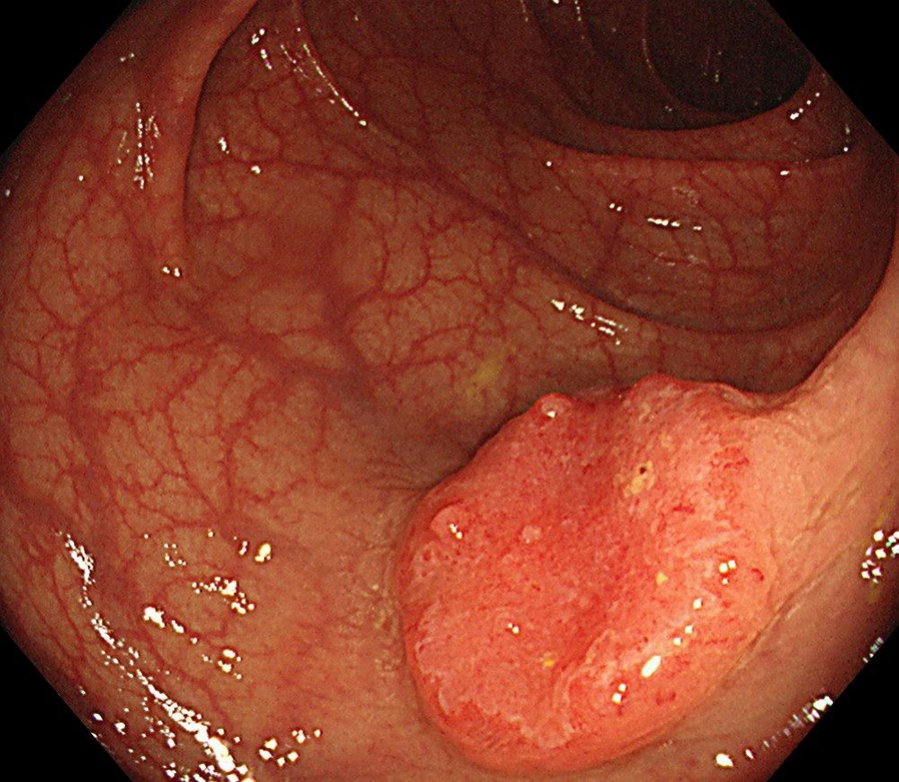
Colonoscopy findings. There is a tumor in the sigmoid colon

However, 28 days before the operation, the patient tested positive for severe acute respiratory syndrome coronavirus 2 (SARS-CoV-2) by reverse transcription polymerase chain reaction (RT-PCR); this test was needed as the patient was a contact person of his wife, who had a fever and was diagnosed with COVID-19. Because the patient was asymptomatic, he was followed up at an isolation facility and was released from isolation 18 days before the scheduled operation. According to the rules of our hospital on elective surgery during the COVID-19 pandemic, he was instructed to wait at home and to observe physical distancing 14 days before surgery. Five days before surgery, a repeat nasopharyngeal swab (NPS) RT-PCR test for SARS-CoV-2 was negative. Considering the previous COVID-19 infection, a chest CT scan and a venous ultrasound of the lower extremity were performed 2 days before surgery. A chest CT scan at this time showed a small ground glass opacity (GGO) in the periphery of the right lower lobe; this was atypical of a metastatic lesion from colon cancer and was not found 2 months before surgery (Fig. 2), thereby suggesting a scar secondary to COVID-19 infection. There were no abnormal respiratory findings upon physical examination, and ultrasound showed no lower extremity venous thrombosis. Therefore, we decided to operate on the patient as scheduled.

**Fig. 2:**
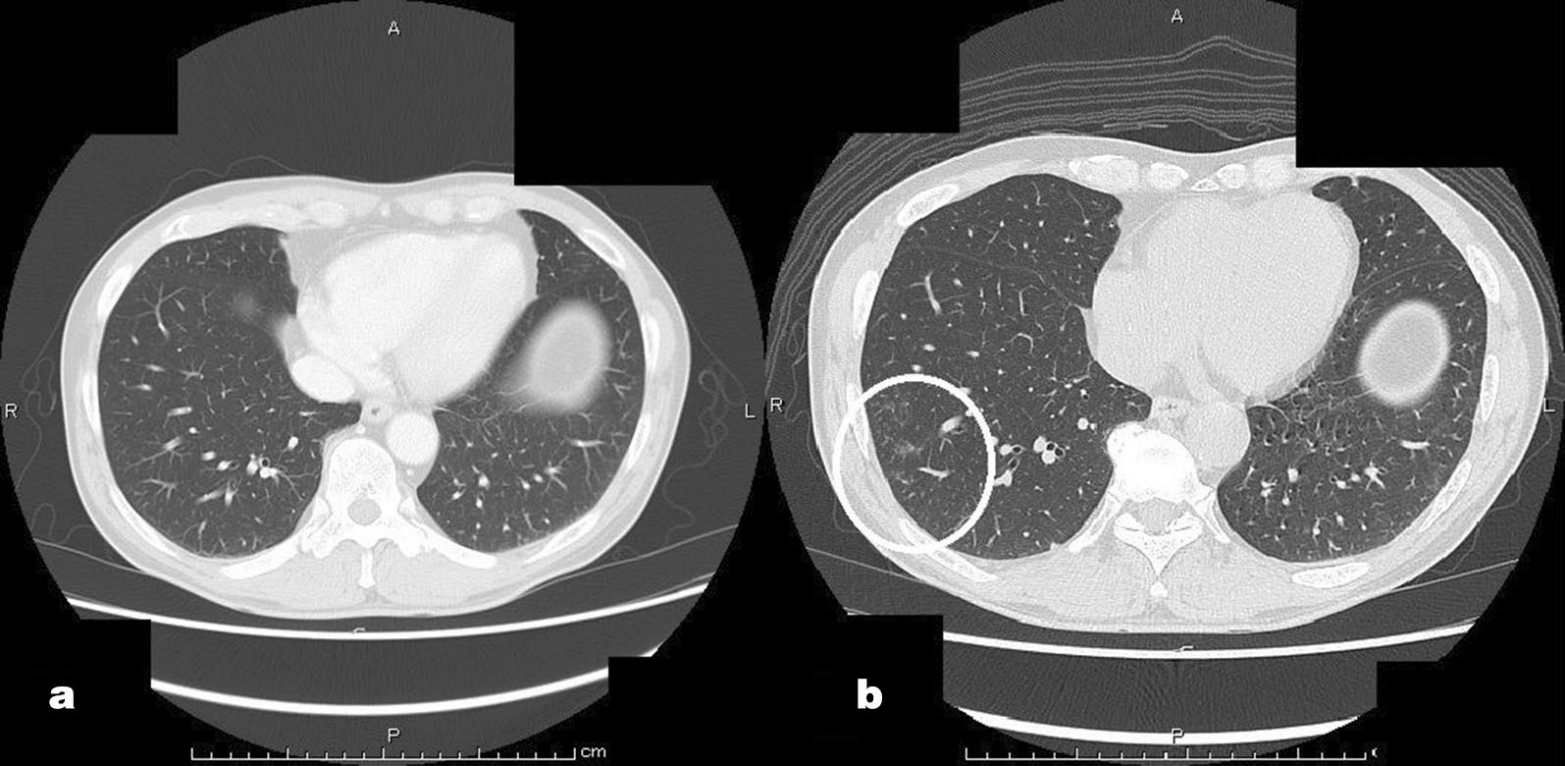
Chest computed tomography scan findings. The images show **a** no abnormal findings 2 months before the operation and **b** a ground glass opacity in the periphery of the right lower lobe 2 days before the operation (white circle)

Laparoscopic colectomy was performed in the usual operating room and not in a negative pressure chamber. Surgeons, anesthesiologists, and surgical nurses wore third-level protective equipment, such as disposable surgical caps, N95 masks, disposable protective suits, disposable latex gloves, and protective glasses, in place of full-face respiratory protective equipment [2]. Tracheal intubation, general anesthesia, and epidural anesthesia were performed as usual. Laparoscopic sigmoid colectomy was routinely performed using carbon dioxide insufflation to cause pneumoperitoneum and laparoscopic coagulation shears. A smoke exhaust device was attached, and frequent suctioning of smoke and mist was performed. Functional-end-to-end anastomosis with an automatic suture device and no stoma was constructed. For perioperative deep-vein thrombosis and pulmonary embolism prophylaxis, elastic stockings and intermittent pneumatic compression were used instead of anticoagulant drugs. The patient had a good operative course and was discharged on the eighth postoperative day. A month after surgery, the patient showed a good course without complications. Two weeks after surgery, the SARS-CoV-2 RT-PCR results of the surgical staff were negative.

## Discussion and conclusion

Considering the high prevalence of COVID-19 worldwide, there is a need to reach a consensus regarding the optimal strategy for elective laparoscopic colectomy in patients with previous COVID-19 infection, considering the safety of both the patient and the medical staff.

In this case, surgery was performed 18 days (within 4 weeks) after the isolation of the patient. According to previous reports, from the viewpoint of safety, surgery is not recommended until at least 4 weeks after the symptoms of COVID-19 resolve [3, 5]; however, this “four-week rule” lacks sufficient evidence because of the small number of cases. Should patients previously infected with COVID-19 have to wait to be operated on for more than 4 weeks? During the COVID-19 pandemic, some cases of elective surgery for colorectal cancer have been needed to be postponed [7], although the resumption of surgical treatment as soon as possible is also necessary [1]. In the present case, surgery was planned relatively early after COVID-19 infection because 2 months had already passed after the diagnosis was confirmed, and the patient was asymptomatic and showed no complications other than mild diabetes mellitus. Even though the preoperative PCR test for SARS-CoV-2 was positive, surgery could be performed at that same time using higher-level personal protective equipment (PPE), to prevent the progression of colorectal cancer.

Although routine evaluation of tolerance for general anesthesia and surgery was performed with blood tests, electrocardiogram, and X-ray, additional venous ultrasound and chest CT scan were needed because of the previous COVID-19 infection. Preoperative screening tests involving venous ultrasound for thrombosis from coagulopathy and chest CT scans for respiratory failure, which are the main causes of postoperative complications in patients infected with COVID-19 [3, 5], were important. It was previously reported that pulmonary complications occurred in 18.5%, 11.7%, and 0.0% of patients who were operated on 1–2 weeks, 2–4 weeks, or more than 4 weeks, respectively, after the resolution of COVID-19 symptoms [5].

Some studies have reported that D-dimer measurement for deep thrombosis may be superior to ultrasound [6] because thrombosis in areas other than the lower limbs may be present. However, high D-dimer may be seen in malignant tumors even in the absence of blood clots [8], which may lead to excessive unnecessary use of anticoagulants. Accordingly, we selected venous ultrasound because it can directly identify deep-vein thrombosis and the needed treatment. If deep-vein thrombosis is suspected by ultrasound, drugs such as heparin can be administered in addition to the usual perioperative anticoagulant therapy. In the present case, only the usual preventive measures were taken because there was no deep-vein thrombosis according to ultrasound.

With regard to chest CT, signs that imply poor lung reserve would dictate strict ventilator management in the intensive care unit and possible delayed extubation after surgery. A previous report [9] advocated the importance of chest CT screening for elective abdominal surgery to determine perioperative management. Despite radiation exposure, it is expected that the spread of the pneumonia image can be identified when no abnormality appears in the blood gas or respiratory function test. In the present case, chest CT showed a small GGO, which was thought to be secondary to COVID-19 infection and had no effect on respiratory function. Therefore, general anesthesia and postoperative management were performed. Currently, there is no consensus regarding the risks of surgical treatment in cases of previous COVID-19 infection. Nevertheless, in asymptomatic COVID-19 cases with no sequelae, such as respiratory dysfunction or deep-vein thrombosis, elective colectomy can be safely performed within 4 weeks after infection. On the other hand, some of the systemic complications caused by respiratory failure and thrombotic tendency may be serious and can be associated with postoperative death. Indeed, the accumulation of additional cases seems necessary for the appropriate selection of patients who can safely undergo surgery within 4 weeks after COVID-19 infection.

Next, we considered the risk of infection to the medical staff. In this case, we instructed the patient to isolate at home for 2 weeks before surgery and ensured a negative NPS RT-PCR test for SARS-CoV-2 5 days before surgery. Previous guidelines specify that surgeons and surgical staff should wear third-level equipment, such as disposable surgical caps, N95 masks, disposable protective suits, disposable latex gloves, and protective glasses, in place of full-face respiratory protective equipment, when operating on COVID-19 patients [2]. In addition, previous reports have indicated that a negative NPS RT-PCR test for SARS-CoV-2 did not translate to a negative test from the intestinal tract or feces [10]. Accordingly, we wore third-level protective equipment to perform the operation. In patients infected with COVID-19, laparoscopic surgery has been reported to increase the risk of infection because of the presence of the virus in the carbon dioxide used to induce pneumoperitoneum and in the mist generated by laparoscopic coagulation shears [11]. On the other hand, the laparoscopic approach may be superior to an open approach because of the shorter time of contact with the abdominal cavity exudates and tissues, and the intact abdominal wall acts as a barrier between the surgical field and the surgical staff [11]. Laparoscopic surgery was selected in this case, considering the advantages of reducing wound pain and the familiarity of the operator. These infection control measures may be the reasons for the subsequent negative RT-PCR tests for SARS-CoV-2 among the doctors involved in this case, although there was a possibility of false-negative results. Further research is needed to confirm this causality.

More than a year has passed since the start of the COVID-19 pandemic. Although the postponement of elective colectomy for colon cancer is unavoidable during a pandemic, it cannot be permanent. At present, strong evidence regarding how to resume elective colectomy is needed. Moreover, as the number of individuals infected with COVID-19 increases, the need for elective colectomy in previously infected patients will be expected. In this case report, we showed that laparoscopic colectomy for sigmoid colon cancer can be successful and safe in an elderly patient who was infected with asymptomatic COVID-19 3 weeks before the surgery. In addition, none of the surgical staff was infected with COVID-19 after the surgery.

We hope that this report will contribute to the accumulation of additional case reports in the future and to the formation of strong evidence on elective colectomy for patients previously infected with COVID-19.

## Data Availability

We agree to share our data.
